# Key issues for participatory research in the design and implementation of humanitarian assistance: a scoping review

**DOI:** 10.1080/16549716.2020.1826730

**Published:** 2020-10-19

**Authors:** Ilja Ormel, Jon Salsberg, Matthew Hunt, Alison Doucet, Lisa Hinton, Ann C. Macaulay, Susan Law

**Affiliations:** a Department of Family Medicine, McGill University, Montreal, Canada; b Graduate Entry Medical School, University of Limerick, Limerick, Ireland; c School of Physical and Occupational Therapy, McGill University, Montreal, Canada; d THIS Institute, Department of Public Health and Primary Care, University of Cambridge, Cambridge, UK; e Institute for Health Policy, Management and Evaluation, University of Toronto, Toronto, Canada

**Keywords:** Accountability, relief work, humanitarian assistance, disasters, NGO

## Abstract

**Background:**

Participatory approaches that engage affected populations are increasingly applied in humanitarian health programs in concert with emerging accountability frameworks and the rapid growth of research in these settings. Participatory initiatives within this domain appear to be largely adopted at an operational level and are infrequently reported as a component of research efforts. Yet the evidence of the benefits of research involving community members is growing worldwide. This is the first review of participatory research (PR) in humanitarian settings.

**Objectives:**

This study sought to understand the extent to which PR values and practices have been adopted in humanitarian health programs and to explore key issues in applying PR in this context.

**Methods:**

This scoping review was based on the approach developed by Arksey and O’Malley. The search for relevant peer-reviewed articles included scientific databases, a humanitarian database, targeted journals and online resources published since 2009. Eleven articles were retrieved and reviewed to identify practices and key issues related to conducting PR in humanitarian settings.

**Results:**

Four key themes were identified: building trust with local research stakeholders and participants; the importance of contextual understanding; implications of collaborating with affected populations in PR, and neutrality of researchers and Non-Governmental Organizations (NGOs). Study teams considered PR as a valued approach where there was mistrust or a need for contextualized understanding. The studies described how adaptations made during the study optimized collaboration with affected populations and how the presence of NGOs influenced the approach and results of PR.

**Conclusions:**

One of the most important contributions of humanitarian health programs is to develop ‘medical practices that are better adapted to the living conditions and priorities of patients who are generally ignored’. Participatory approaches, such as PR, support the development of health-related practices that are more relevant and sustainable for affected populations.

## Background

The use of participatory research (PR) methods in humanitarian health programs remains limited despite the increasing application of participatory approaches at an operational level, emergent accountability frameworks and the rapid growth of research in humanitarian settings. Yet the evidence of the benefits of research involving community members is growing worldwide.

### Participation of affected population in humanitarian response

Humanitarian health programs – where organizations and individuals respond to the health needs of populations affected by humanitarian crises such as armed conflicts, natural disasters or epidemics – are facing challenges that are growing in scale, scope and complexity [1]. The average duration of such crises has increased from four to 7 years between 2005 and 2017 [2]. Major crises in the past (for example, the 1994 Goma refugee crisis, 2004 South Asian Tsunami, 2010 Haiti earthquake and 2014/2015 Ebola epidemic in West Africa [3] have generated new insights and concerns about the effectiveness and value of humanitarian assistance [4–6]. Aid organizations have more recently focused on the notion of ‘accountability’ of their work with respect to local populations [7,8], with initiatives such as the Core Humanitarian Standard (CHS) and the Active Learning Network for Accountability and Performance (ALNAP) who have promoted greater participation of affected populations. There is, however, not yet a single-accepted definition for ‘accountability’ in the humanitarian sector [3]. Accountability can now be assessed in consideration, for instance, of how donor money was spent, but also how humanitarian programs respond to the needs of affected populations. Accountability towards affected populations alone can mean different things: empowering aid recipients; being in an optimal position to do the greatest good; meeting expectations; and, being liable [3].

### Research in humanitarian settings

Another factor that plays an important role in the design and implementation of humanitarian health programs is state of evidence underpinning current humanitarian practice [9,10]. While research in humanitarian settings has significantly increased in the last decade [10] there is still a high need to identify and address current evidence gaps even in settings with limited funding and high immediate survival needs [11]. Health research in these settings typically aims to contribute to more effective humanitarian health programs, optimisation of the delivery of care in crisis settings and the production of knowledge that is appropriate for these specific settings [11]. Knowledge created through research informs and influences humanitarian health programming, but it must be kept in mind that ‘what a researcher finds out is inherently connected with how she finds it out’ [12]. The nature of the design, implementation and analysis will influence the process of knowledge generation. Humanitarian health programs are typically implemented in complex settings, often assisting vulnerable and marginalized population groups. Health researchers conducting research with marginalized or vulnerable population groups stress the importance of using PR approaches with populations who have historically been left out of the research process [13,14].

### Participatory research (PR)

PR covers a wide range of different terms including, among others, community-based participatory-research (CBPR), participatory action research (PAR), integrated knowledge translation (where decision-makers collaborate in the research process with the aim to enhance the relevance and use of research [15]) as well as co-design or co-production initiatives [16]. Community engagement (where collaboration is established between researchers and communities) is one of the approaches that falls under a participatory approach [16]. PR requires meaningful involvement of end users that can occur across the range of research activities throughout the life cycle of a project or initiative, including engagement in defining the research question, collecting and interpreting data, and reporting and applying the findings [17,18]. Recent reviews of PR approaches argue that engaging people as partners in the co-construction of research [17] increases the commitment of these stakeholders to use the research findings and take action [19] and improves the relevance of research findings [16].

### Participatory research in humanitarian settings

While participation, accountability and research are now an integral part of humanitarian programming and implementation, there remains a lack of insight and evidence regarding the extent of PR approaches applied in humanitarian health programs. Critical reviews of PR approaches in predominantly Western health-care settings sought to distil the key challenges and added value of PR [19], mechanisms ‘by which PR adds value to the research process’ [17], and ‘successful strategies to engage research partners for translating evidence into action in community health’ [20]. Previous reviews of PR approaches have predominantly focused on research conducted in Western settings whereas this review focuses on PR in humanitarian settings. As this is a novel undertaking, we adopted a scoping review methodology. Our research question is broad and exploratory in nature, aiming to ascertain the range of different study designs that have been deployed in this setting and associated findings, in contrast to the approach adopted in systematic reviews that typically focus on a well-defined question where appropriate study designs can be identified in advance [21].

## Methods

Scoping reviews are typically conducted to map key concepts in a research area or summarize the main sources and types of available evidence [22,23]. This approach can be particularly useful in an area that is complex, has not been reviewed comprehensively before [23] or in areas with emerging evidence [24]. In the case of PR in humanitarian settings, where there is inherent complexity related to context as well as novelty in the adoption of participatory approaches within research, we believed that a scoping review could provide insight into the state and scope of existing evidence. We adopted the specific approach advanced by Arksey and O’Malley [21] that involves five key steps: identifying the research question; identifying relevant studies; study selection; charting the data; and, collating, summarising and reporting the results. We worked closely with a qualified health sciences librarian who has extensive experience in conducting scoping reviews. We used the Preferred Reporting Items for Systematic Reviews and Meta-Analysis (PRISMA) criteria to guide the conduct and reporting of the review [25].

***1. Identifying the research question***

The research question was developed iteratively with the research team and librarian in the preliminary phase of scanning the literature and establishing the search strategy: *How, and to what extent, have PR values and practices been described in published articles of humanitarian health research studies?* Our secondary question was: *What are key issues related to applying PR in humanitarian settings as reported in these studies.*

We considered the literature in relation to three concepts: ‘PR’ and ‘humanitarian settings’ and ‘low- and middle-income countries (LMICs)’. The search terms for these three concepts have been adapted from earlier published reviews: a scoping review of integrated knowledge translation in evaluations in health care [16]; a realist review of studies describing PR partnerships [17]; an evidence review of research on health interventions in humanitarian crises [10] and, the LMIC search filter developed by the Cochrane Collaboration in 2012 [26] and the LMIC country list published by the World Health Organization (WHO) [27]. Please see Appendix A for a detailed overview of all the search terms.

***2. Search strategy***

A preliminary search was undertaken with the help of a qualified librarian. The results of this search were discussed with team members and used to establish a more comprehensive search strategy with explicit inclusion and exclusion criteria (see Appendix B) and multiple information sources (see Appendix C). First author (IO) conducted all searches in close collaboration with author SL and a qualified librarian. The search was conducted from May through to September 2017 and the search was updated again for articles up to 1 February 2019. This phase yielded 3729 titles.

***3. Identification of relevant studies***

The initial search yielded a large number of irrelevant articles, for example including articles on health emergencies in high-income countries, emergency preparedness and disaster prevention. This reflected the challenge in defining the terms and concepts related to humanitarian settings and PR. In collaboration with the librarian, more precise and selective search criteria were developed (e.g. war$.mp was replaced with war1$.mp). In addition, it was decided to add search terms for LMICs and to limit the search to studies published after 2008, covering a period of 10 years which was deemed to be likely to capture most relevant literature given that PR approaches in humanitarian settings are a relatively new practice.

In the next phase, we selected relevant studies and refined the inclusion and exclusion criteria through initial screening of titles, abstracts and then review of full articles. See Appendix C for a more detailed description of the exclusion and inclusion criteria as well as the scanning process.

***4. Charting the data***

We reviewed each article to extract relevant data [including bibliometric data (e.g. title, publication year, authors), reasons for PR, challenges and limitation, outcomes] and identify themes in response to the primary and secondary research questions using a qualitative descriptive analysis [28].

***5. Collating, summarising and reporting the results of identified data***

IO and SL first coded all relevant texts independently and we then examined patterns and linkages within and across articles to identify themes which reflected key issues addressed in the data. This process was iterative where we constantly returned to the articles to read certain sections again while coding and selecting text abstracts.

### Results

The outcome of the review is twofold: firstly, the scanning of relevant literature resulted in eleven articles. This finding reflects the relatively limited extent where PR values and practices have been adopted in research within humanitarian health settings. Secondly, after close reading of the eleven articles based on eight studies, we identified four themes representing important key issues while applying PR in humanitarian settings: building trust with local research stakeholders and participants, importance of contextual understanding implications of collaboration with affect populations in PR and, interdependence between PR and the role of NGOs (see Table 2 for a detailed description of the findings).

**Table 1.** General information of identified studies

1. Author (year) 2. Study location and context	1. Title 2. Type of PR (type of framework)	1. Study objectives 2. Health Focus
1. Abdulrahim et al. (2010) [35] 2. Palestinian refugee camp in Lebanon (Beirut)	1. The potentials and challenges of an academic–community partnership in a low-trust urban context. 2. CBPR	1. Designing and implementing an intervention to improve the mental health and enhance school attachment. 2. Mental health
1. Afifi et al. (2011) [36] 2. Same as in Abdulrahim (2010)	1. Developing a logic model for youth mental health: participatory research with a refugee community in Beirut. 2. Same as in Abdulrahim (2010)	1. Planning, implementing and evaluating a logic model and intervention 2. Same as in Abdulrahim (2010)
1. Makhoul et al. (2014) [39] 2. Same as in Abdulrahim (2010)	1. Community-based participatory research in complex settings: clean mind-dirty hands. 2. Same as in Abdulrahim (2010)	1. Same as in Afifi (2011) 2. Same as in Abdulrahim (2010)
1. Jones et al. (2018) [37] 2. Post Ebola response in Liberia	1. Rebuilding people-centred maternal health services in post-Ebola Liberia through participatory action research. 2. PAR	1. Build communication between stakeholder groups and identify impacts of Ebola epidemic and shared actions to improve the system. 2. Maternal health
1. Tanabe et al. (2017) [32] 2. Refugee settings in Kenya, Nepal, Uganda	1. “Nothing about us, without us”: Conducting participatory action research among and with persons with disabilities in humanitarian settings 2. PAR	1. Identify specific risks, needs, and barriers to access sexual and reproductive health (SRH) services, and the capacities and practical ways in which these challenges can be addressed. 2. Sexual and reproductive health
1. Elmusharaf et al. (2017a) [40] 2. Post war, South Sudan (Renk County)	1. Participatory Ethnographic Evaluation and Research: Reflections on the Research Approach Used to Understand the Complexity of Maternal Health Issues in South Sudan. 2. PEER (interpretivist approach)	1. Provide a contextualized understanding of maternal health issues in South Sudan and provide recommendations for programmatic health interventions. 2. Maternal health
1. Elmusharaf et al. (2017b) [33] 2. – Same as in Elmusharaf (2017a)	1. Social and traditional practices and their implications for family planning: a participatory ethnographic study in Renk, South Sudan. 2. Same as in Elmusharaf (2017a)	1. Gain in-depth understanding of the social determinants of family size to inform local policy and practice. 2. Same as in Elmusharaf (2017a)
1. Glass et al. (2012) [34] 2. Post conflict, Democratic Republic of Congo (DRC)	1. A Congolese-US participatory action research partnership to rebuild the lives of rape survivors and their families in eastern DRC. 2. PAR	1. Understanding the health, social, cultural and economic factors that influence reintegration to families and communities. 2. Rape
1. Edstrom (2018) [38] 2. Refugees from Great lakes area, Uganda.	1. Breaking the Spell of Silence: Collective Healing as Activism amongst Refugee Male Survivors of Sexual Violence in Uganda 2. Collaborative and grounded approach	1. Explore how male refugee survivors of sexual violence have been able to organize, heal and become activists. 2. Healing from sexual violence against men
**Not all inclusion criteria but relevant**		
1. Nelems and Curie (2012) [30] 2. Refugee camp, Jordan	1. Listening to Iraqi refugee children in Jordan, but then what? Exploring the impact of participatory research with children. 2. PAR	1. Understand lived experiences and explore the potential for PR to transform programming and the obstacles to institutionalising change. 2. General protection
1. Shanks et al. (2015) [29] 2. Violence, Chechnya and conflict, DRC	1. Losing the tombola”: a case study describing the use of community consultation in designing the study protocol for a randomised controlled trial of a mental health intervention in two conflict-affected regions. 2. Co-design	1. Consultation with community prior to finalising RCT study protocol 2. Mental health

**Table 2.** Findings with regards to the four themes; building trust with local research stakeholders and participants, the importance of contextual understanding, implications of collaborating with affected populations, neutrality of researchers and NGOs

**Reported findings – Building trust with local research stakeholders and participants**	
Reasons for mistrust	Competing for funding, little return from research, research approach, overall mistrust towards the health care system, organisations and NGOs
Value of PR	Contributed to improved trust, was seen as a way to support trusted health systems and could help reduce the required time for trust building
Helpful approaches	Smaller and more confidential meetings, funding to include requirement for collaboration, transparency, allowing time to build trust, include community in decision making
Considerations	Pay attention to mistrust between collaborators, contextualize mistrust within broader structural conditions, allow sufficient time for trust building, community trust is as important as participation
**Reported findings – Importance of contextual understanding**	
Impact context	The complexity of the settings impacted the level of participation and application of research methods, population prioritised immediate benefits over longer term benefits, context challenged implementation of project recommendations, presence of research fatigue,
Importance context	A contextualized understanding can help tailor interventions that are more likely to be accepted and utilised, collaborators need to be able to respond to changing contexts, important to consider health issues as situated in and linked to social contexts – this requires the use of multi-methods to understand reality
Value of PR	Increased understanding of complex issues and hard-to-reach communities, complexity requires interdisciplinary teams, local experience and knowledge can help overcome cultural barriers
Helpful approaches	PR approaches require time, commitment, and top-down complimentary support to be of maximum benefit, funding that supports assessing the complexity,
Considerations	Have attention to and address context specific challenges and community forces that influence participation, important to take time to understand community perceptions to help localize the study, participatory approaches can support more resilient, responsive, and trusted health systems.
**Reported findings – Implications of collaborating with affected populations**	
Challenges	Engaging of male participants was difficult, camp settings interfered with participation, limited participation because of camp conditions,
Value of PR	Allowed for direct local consultation, minority groups were heard, member commitment sustained partnerships, community gained confidence to act in their health system and changed perception on actions women could take, possibility for new learning and development of new approaches
Helpful approaches	Flexibility in applying research methods (e.g. consent process, methods, recruitment or objectives, allow informal participation, use of symbols and short interviews for illiterate data collectors, adaptations to language use and translations, sufficient time at start, broad community representation
Considerations	Assess feasibility of participation at start-up, assess forces that affect participation, identify priorities of stakeholders, promote ownership over findings, identify local networks, evaluate (ethical) risks of study designs, don’t expect participation in all phases, need for further research on how to include hard-to-reach populations, attention to capacity building, reflective practices and genuine dialogue
**Reported findings – Neutrality of researchers and NGOs**	
Role NGOs	NGOs facilitation of research may have influenced discussions, biased project trajectories or marginalised certain stakeholder groups, PR efforts needed to run parallel with NGOs actions, researchers perceived competition with NGO, stressful early meetings due to competition for funds amongst NGOs, people, affiliated with NGOs, felt compelled to take part
Value of PR	PR inspired community to be part of research, outcomes helped other NGOs to prioritise projects based on children’s perspectives, organisations became convinced of value of participatory engagement, approach was feasible despite the context and provided valuable information
Helpful approaches	NGOs working alongside co-facilitators from stakeholder groups to minimise impact of presence NGO workers, NGO leading research sessions helped to clarify any misperceptions, funding to support collaborative approaches and not only fund evaluations of outcomes,
Considerations	NGOs need to find new ways of working better to respect local knowledge and experiences, be more flexible in programming based on children’s priorities, need for longer-term staff positions, NGOs wanted to be engaged but found various aspects difficult,

### Search results

After the removal of duplicates (n = 191), the academic databases produced 951 articles. These were screened together with 2135 articles identified in the MSF database and 643 titles extracted from the humanitarian organizations’ websites. The 3729 records were screened by title by one reviewer (rejection of 3603 articles) and abstract (rejection of another 99 articles). The abstract and original text of 37 articles were then independently assessed by IO and SL to select the final sample. Discrepancies between the researchers were resolved by discussion. In total, we selected 11 articles for inclusion in the review. See Flowchart 1 and Table 1.

**Flowchart 1.**
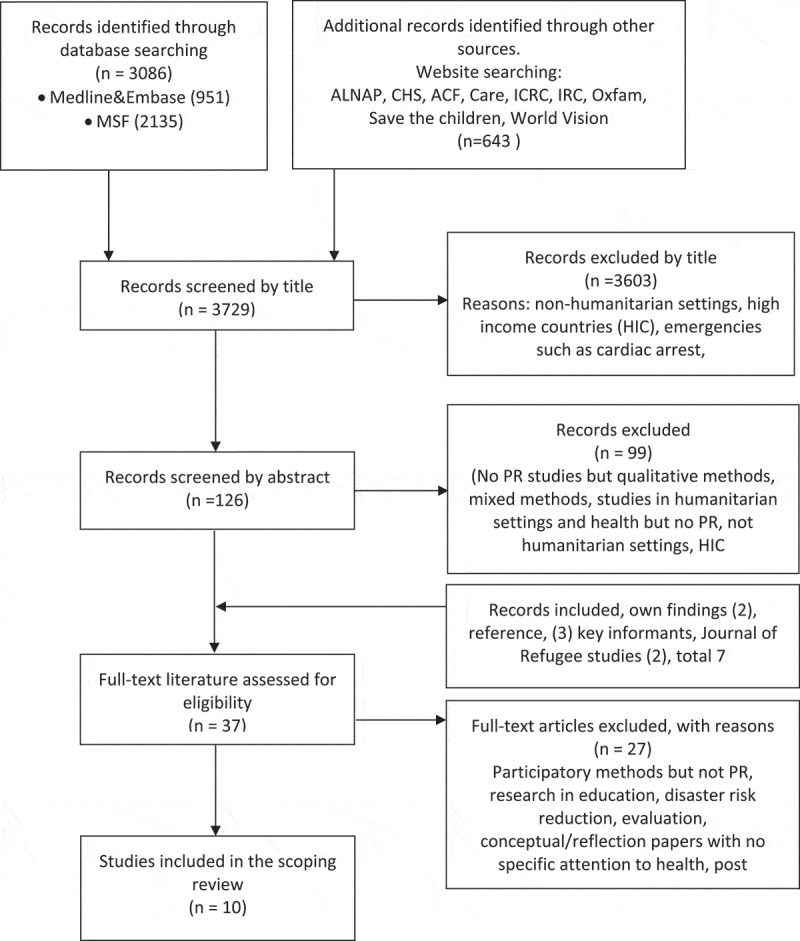
The process of article selection for the scoping review

The 11 identified articles were based on the results of eight research projects. The search yielded two studies that we initially considered excluding. One study consulted and engaged with the community to inform the design of a Randomized-Controlled Trial (RCT) [29] which we identified as one of the first stages of participation. A second study did not have a precise health focus but related to health in that it focused on child protection where there were mental health implications for the participants [30]. We decided that these articles contained relevant information and could contribute to a better understanding of the value of PR in humanitarian settings. Half (50%) of the studies were published in the last three years, which is consistent with the growth of research studies in humanitarian settings [31] and may indicate an increase as well in PR in humanitarian settings. The studies covered a wide variety of countries, different humanitarian crises, a range of participatory approaches and different health foci. The majority of studies aimed to better understand risks, needs, and barriers in relation to health, social, economic and cultural factors, as well as lived experiences. These studies provided practical ways or recommendations in which these kinds of issues could be addressed [30,32,34,38]. Two studies aimed to design and implement a program to improve current health services available in the humanitarian setting [35–37]. A study in Uganda analysed data, obtained through collaborative approaches, to improve the understanding of how a local group for male rape victims became organized [38], and lastly a study conducted in the Democratic Republic of Congo (DRC) and Chechnya aimed to consult with the community prior to finalizing an RCT [29].

### Building trust with local research stakeholders and participants

Trust was the primary reason as expressed by authors why several studies adopted a PR approach, while for other studies improved trust was described as an outcome in contexts with existing mistrust between communities and humanitarian organizations or amongst local actors. One study described how participation helped to adapt the study in such a way that it would not create mistrust [29].

A number of studies identified the motivation for their choice to apply PR as a way to build up trust amongst the community members and actors in the health system. The IRC, for example, partnered with a local hospital in Liberia when Ebola incidence started to decrease in late 2014. The slow rate of service use uptake post-Ebola was anticipated to be caused by fear of infection and mistrust of health-care professionals [37]. Through a PAR approach, IRC was able to engage with local-trusted health personnel that contributed to more meaningful forms of community and health worker participation. Jones et al. have argued that this can support the development of more resilient, responsive and trusted health systems [37]. While trust itself was not specifically measured it is noted in the article that the PAR approach resulted in strengthened relationships and improved communication. Similarly, in South Sudan collaborating with locally trained data collectors enabled the team to reduce the time required for data collection and trust-building and helped overcome trust issues between the North Sudanese researcher and South Sudanese study participants. And in Lebanon, the PR process contributed to reduced mistrust amongst NGOs and United Nations (UN) agencies due to strengthened relationships [35,36]. These authors noted the importance of contextualizing mistrust ‘within the broader structural conditions that create conflict and competition between partners’ and the importance of building on community strengths. For the authors of this study, it was evident that ‘respect and trust are as, if not more, important, than participation at all stages of the research [39]. Even though the PR process had improved trust slowly, in this study, it had not completely dissipated mistrust [35]. The participatory process in a community consultation study to inform the design of an RCT study revealed that affected populations distrusted research conducted by NGOs as they feared a hidden purpose. In addition, this community suggested that all questions in the survey should relate to the illness otherwise it would be seen as spying [29]. Shanks [29] reported that splitting up existing community groups (e.g. religious leaders, health-care staff members) for focus groups would have led to distrust, contrary to finding from Abdulrahim et al. [35] where trust in their collaborative meetings was enhanced by forming smaller youth groups.

### Importance of contextual understanding

The articles described how the complexity of the humanitarian settings (e.g. political restrictions and displacement) and/or of the particular health issues (e.g. rape victims and Ebola outbreak) negatively affected the level of participation, required adaptations to PR methods or created obstacles for participation.

In the study in Lebanese refugee camps, the level of participation was affected for men who had limited time to participate given they were often working more than one job, but also because of the outbreak of a war and difficulties in setting up participatory approaches due to the United Nations Relief and Works Agency’s (UNRWA) bureaucracy [39]. Populations also expressed a lack of interest in participating, as the data collected by international NGOs were not going to be used to implement and sustain programs [34,39].

On the other hand, it was also noted that PR approaches, in these complex settings, contributed to a better contextualized understanding which helped to address health issues and develop more effective, acceptable and tailored humanitarian services. PR contributed to a better understanding of the complex context in South Sudan where a maternal health program was being implemented. Addressing barriers to maternal health involves changing complex behaviours; a good understanding of the context of (hard-to-reach) communities and the complexity in which different behaviours occur [40] enables the tailoring of services so that they are more likely to be accepted and used [39,40]. A study of an approach to rebuild the lives of rape survivors and their families in eastern DRC demonstrated that different factors (e.g. security, social norms, and economic impact) have negative impacts on health and human rights of the local population. Addressing health therefore requires an interdisciplinary collaboration with diverse sets of expertise, including health care and gender specialists, human right lawyers and military advisors [34].

Furthermore, in certain studies, the authors noted the link between contextual factors and the level of impact or outcomes of the initiative or program. It was, for example, reported that the complexity of the context (factors such as the lack of staff continuity in organizations and the institutionalized power relationships between the Jordanian government and the donor community members) hindered the implementation of the PR results [30]. On the other hand, however, the PR results helped organizations form a better understanding of the context and influenced how one NGO related to their staff and volunteers in more egalitarian ways ‘based on a greater understanding of the complexity of their lives’ [30].

### Implications of collaborating with affected populations in participatory research

In most studies, collaborating with affected populations as part of the participatory process involved engagement by researchers in training local partners, and further, that collaboration led to changes to the PR question, the methods or the co-development of methods with the affected population.

Several studies described substantial training efforts for local partners over 3–4 days [32,35,37,40]. It was noted that collaborating partners appreciated the certificate awarded at the end of the training as well as the opportunity to learn [40]. In Nepal, the importance of participatory and co-production processes were highlighted when data collectors invited family members to join a debriefing session as it was seen as an important personal achievement to have a critical role in a research process [32]. The attention to capacity building enhanced participation, reflective practices, and created conditions for genuine dialogue [40].

Participation of local *data collectors* was sometimes made possible through adaptations of the approach, which brought certain benefits. In South Sudan, interview guidelines were developed with the use of symbols and drawings (developed with the collaborating partners) so that illiterate data collectors were able to conduct the interviews [40]. In another study, data collectors who had disabilities experienced difficulties with the limited accessibility infrastructure of the camp yet had the ability to engage and relate to participants with disabilities while ‘creating a safe and open environment for dialogue’. In addition, their presence served as a powerful icebreaker [32]. Engaging local women as researchers in South Sudan helped to identify women who were willing to participate and enhanced their willingness to trust the researcher [40]. It also helped gain more in-depth understanding and insightful information [40]. It was also noted that partners were keen and able to take active roles in research [38] and their high level of commitment to enhancing youth welfare served as a driving force to sustain the partnerships [35].

Some studies described making changes to the design, data collection and recommendations such as allowing short conversations with vulnerable individuals who otherwise would not have been included [32] or by allowing multiple participants rather than individual participation so that people were more confident to participate [37]. PR also opened up new learning and the development of new approaches [38] as well as the application of a rights-based approach which fostered increasing levels of participation [32]. In Lebanon, the study group felt at times challenged as the impoverishment and the difficult living conditions in the camp influenced the participants’ interests in the direction of research that offered immediate benefits (e.g. providing English lessons) over scientific health research that offered no immediate benefits. These authors advised that it is important to assess the feasibility and level of interest to participate when starting a participatory research project to understand barriers and possible strategies to mitigate them [39]. Finally, it was suggested that there is a need for further research to look at how to best include the perspectives of hard-to-reach communities [40].

One study reported that none in the collaborating community received compensation due to a lack of available funds [35]. Two studies described providing a small token of appreciation for the *participants* such as monetary or non-monetary gifts/contributions (small food items or money) as well as the provision of refreshments and reimbursement of transportation costs [29,37]. Another study assessed what the community felt should be the compensation for participation and participants advised that a small amount of food or money would be appropriate, but felt that should not be too much in order to prevent participation solely for the incentive [29]. The other studies did not mention whether or not partners were paid or compensated in any way. Difficulties with participation were also noted due to the challenging working conditions and the need to prioritize paid work over volunteer participation [39].

While most studies mentioned the specific collaborating partners (e.g. international and local NGOs, universities, representatives of UN, health-care workers) it was not always clear in the published reports exactly which collaborators took part in each of the various phases of research, such as in the analysis, interpretation and reporting of the data. In addition, only two articles included local partners as authors [34,37].

### Neutrality of researchers and NGOs

To enable PR in humanitarian settings, researchers are often dependent or reliant on the structures and processes of NGOs to enable certain research processes due to security issues, access [12] and the capacity of staff members to conduct research in these kinds of settings. How the research is going to be conducted, and how the research and researcher are perceived in the field are therefore also influenced by the work and reputation of the NGO. Several authors reflected on how collaboration with the NGOs influenced research studies. In some cases, the research in the selected articles was led by a representative of an NGO, in other cases, external researchers depended to some extent on the collaboration with NGOs to be able to conduct their research. This interaction had both potentially positive and negative impacts on the research.

Some collaborating NGOs also provided the services that were subject of the research and this dynamic potentially had an impact on the data collection and results [29,37,39,40]. In Lebanon, participants felt compelled to take part as their families were affiliated with the NGOs [39]. In Congo and Chechnya, it was anticipated that this situation biased responses to be more positive in an attempt to please or not offend the NGO staff, but at the same time, it was also identified as a strength as they had the opportunity to address more directly any misconceptions about the programme and the NGOs’ objectives [29]. In Liberia the authors describe that this may have influenced ‘the discussions, biased project trajectories or unintentionally marginalised certain stakeholder groups’ and PR efforts needed to run parallel with the institutional actions and resources [37]; in order to minimise these effects, the NGO worked alongside co-facilitators from across the stakeholders groups [37].

The presence of NGOs, not related to the study, also influenced the PR approach. In South Sudan, the team was only able to offer participation in the research project and therefore anticipated difficulties recruiting partners whereas other NGOs were offering food and services for participation in a variety of activities. PR was identified as an approach that could inspire and motivate the community to be part of the research [40]. In Lebanon, the funding structure in the camps created an atmosphere of competition and mistrust amongst the NGOs, rather than a culture of collaboration. The researchers recommend that funding agencies need to improve community participation and collaboration by setting up funding structures that promote collaborative processes [39].

The collaboration and results of PR also had a positive impact on other NGOs. One study specifically focused on exploring ‘the potential for PR to transform programming and the obstacles to institutionalising change’. One of the most profound and unexpected outcomes was that participating NGOs became convinced of the value of participatory engagement to better understand the local context. These NGOs developed a greater commitment to participatory approaches, felt better equipped to publicly discuss the findings based on the availability of ‘sound evidence’ and changed their advocacy approach [30].

## Discussion

This scoping review was conducted to better understand key issues in relation to PR as designed and implemented in humanitarian settings. We have demonstrated that the use of PR approaches in humanitarian settings positively contributed to improved trust, a better understanding of complex issues, and engagement with vulnerable and marginalized populations. While participation, accountability and engagement gain prominence in the policy and strategy documents of humanitarian organizations, this review demonstrates that participation needs more than just the rhetoric. The results of the scoping review indicate that PR requires a considerate approach, interdisciplinary teams, time, and flexibility to adapt the methods and tools to the local context. In addition, it is important to continuously consider and address issues such as tense relationships, neutrality, complexity and trust.

The included studies in this review contained only sparse information related to compensation provided to collaborating stakeholders. At the moment, there is no clear guidance on whether collaborators should be paid (and if so, how much and when), with proponents arguing that participation costs time and should therefore be compensated while others argue that payment may negatively impact natural collaborative systems and reduce the willingness to volunteer to support local initiatives [41]. In addition, the level of collaboration with key stakeholders is frequently not clearly described in the identified studies. This is a common critique of published PR studies and approaches [42,43]. This lack of recognition for the contributions of local partners in research may be indicative of the continued challenges with existing inequity and asymmetrical power relations within such collaborations or partnerships [44].

There are countless publications, books and reports urging for change in the way humanitarian assistance is provided. These approaches to change and the paradigms underpinning such approaches are likely ‘bound to be partial and incomplete and shaped by the author positionality’ [45]. Within the humanitarian innovation field, it is noted that there is ‘a longstanding and unjustifiable lack of engagement with recipients of aid’ [1]. In the book entitled ‘*Time to listen: hearing people on the receiving end of international aid’*, Anderson reports on the insights, ideas, and analysis of almost 6000 people who received humanitarian assistance. Affected populations indicated that they want a system ‘that integrates the resources and experiences of outsiders with the assets and capacities of insiders to develop contextually appropriate strategies for pursuing positive change’. This suggests a need to move away from the current approach with a dominant focus on the delivery of resources (and knowledge) towards a system that supports a more collaborative approach to the analysis of the context, design and decisions about the best strategy [41]. While the need to move away from top-down systems is now broadly acknowledged, it remains challenging to transform humanitarian health programs.

The four themes discussed in the results are not about what could be described as the ‘hardware’ of humanitarian assistance – or the delivery of resources. We describe in our findings what is ‘in between’ humanitarian structures and systems, such as the interpersonal relationships, trust, collaboration and the intersections between components of what are characteristically complex contexts – the ‘software’. It is argued that the ‘software’ has a major influence on the success and effectiveness of humanitarian programs [46,47]. If humanitarian organizations truly want to build trusting relationships with affected populations and move beyond a top-down approach of the implementation of evidence-based knowledge then there is a need to engage affected populations at all levels of humanitarian assistance, including research in this field. PR is one of the approaches that contributes to a better understanding of complex systems while generating new knowledge in a mutual learning process.

### Implications

Research conducted in humanitarian settings is often limited in the types of studies that can take place [48] given the context and constraints in humanitarian settings. This is not unique to PR, engaging vulnerable or hard to reach populations remains an issue in many different areas of PR but much can be learned about effective engagement practices by comparing the literature, for example, from PR research experiences in the fields of HIV [49], women discharged from prison [50] and mental health research [51] among others. The studies included in this scoping review provided some insight into how PR was applied, the challenges, and indications of the value of such approaches in humanitarian settings. See Table 3 for implications and recommendations.

**Table 3.** Implications and recommendations for future research derived in this scoping review

Type and source of recommendation	Suggested areas for further work
Author recommendations for others involved in assessments of HP programs	explore for the availability of guidelines and training resources with regards to PR in humanitarian settings consider standard ways of reporting on incentives, honorariums, role of the collaborating partners in the data collection and analysis, and reporting on the continuation of research studies Include local partners on articles, presentations and reports.
Suggestions for future research	explore timing and conditions for when it may be appropriate and meaningful to apply PR approaches in emergency responses further testing and adaptation of PR approaches in humanitarian settings improve methods or approach to understanding the context and complexity of humanitarian health programs (better descriptions of context and setting for programs and studies) Engage with affected populations as well as with key decision-makers such as health-care professionals, policymakers, government agency leaders to help identify problems (and shared understanding of the problems), and improve implementation of results into practice, using, for example, an integrated knowledge translation approach.
Recommendations drawn from included articles	increase funding opportunities that encourage participatory approaches or support research on the processes of PR rather than on evaluation and outcomes [39] address the scarcity of bottom-up health system research approaches [40] increase commitment to learning across the humanitarian community [30] document more of the PR experiences in humanitarian crises settings [39].

### Strengths and limitations

This scoping review was based on a search of various types of resources (academic databases, NGO websites, references from key interviews, journal searches). The selection process for the articles was conducted in close collaboration with a second researcher and reviewed in discussion with an expert advisory committee. There were important limitations in this review. Our search was limited to two academic databases given the likely yield based on our preliminary exploratory searches; we also reviewed references within documents, as well as reports and publications on NGO websites. We did not, however, include a full review of unpublished or ‘grey’ literature, nor did we search in languages other than English. Future reviews could consider the inclusion of other databases or grey literature to increase the number of identified articles, provide additional information and lessons [52] from PR research and to include consultations with community members and key stakeholders to validate the scoping review findings [24]. The search terms PARTICIPATORY RESEARCH and HUMANITARIAN CRISES are difficult to define but we optimized our search strategy as guided by other published strategies [10,16,17] and by adding search terms for ‘LMIC’. Future searches could consider checking whether the terms community engagement, pandemic and endemic provide an additional yield of relevant papers. Further development of effective search strategies and common definitions to support such work in the future would strengthen the methods and findings for such a review with further lessons for PR and humanitarian programs.

## Conclusions

One of the most important contributions of humanitarian health programs is to develop ‘medical practices that are better adapted to the living conditions and priorities of patients who are generally ignored’ [53]. Learning how to optimize and improve humanitarian health programs for those that are deprived of access to health care, requires the inclusion of these populations throughout the processes related to the production and application of new knowledge. PR approaches offer potentially effective mechanisms for identifying priorities for change, adapting medical practices to the local context, improving trust and engaging vulnerable and marginalized populations or community groups in sustainable solutions.

## Paper context

In the provision of humanitarian aid, engagement with local communities and affected individuals is largely reported an operational level, and infrequently within research. This is the first review of participatory research (PR) in humanitarian contexts, which aims to contribute to the growing evidence regarding the benefits of research involving end-users. Those involved in humanitarian aid should consider PR approaches that involve the co-production of new knowledge in collaboration with affected populations for relevant, sustainable solutions.

## Appendix A. Search terms for scoping review in databases MEDLINE and Embase

**Table:**

Concept 1 – PR or IKT	2 – Humanitarian crises	3 – LMIC
Patient Participation/	Humanitarian$.mp.	Developing Countries.**sh,kf.**
Engag* adj3 patient*.mp	Relief work/	(Africa or Asia or Caribbean or West Indies or South America or Latin America or Central America).**hw,kf,ti,ab,cp.**
Participatory research.mp.	relief work.mp.	
Interactive research.mp	Disaster$.mp.	
Action research.mp.	Disaster medicine/	((developing or less* developed or under developed or underdeveloped or middle income or low* income or underserved or under served or deprived or poor*) adj (countr* or nation? or population? or world)).**ti,ab.**
Social responsibility/	Disasters/or	
Accountability.mp.	Disaster planning/	
Participative research.mp	Disaster victims/	
Participatory rural.mp	(Emergency health care or emergency healthcare).mp.	
Participatory appraisal.mp	Armed conflicts/	((developing or less* developed or under developed or underdeveloped or middle income or low* income) adj (economy or economies)).**ti,ab.**
Emancipatory research.mp	Conflict$1.ti	
Empowerment evaluation.mp	Armed conflict$.mp	
cbpr.mp	Refugees/	
Collaborative inquiry.mp	(refugee$ or evacuee$ or evacuated).mp	(low* adj (gdp or gnp or gross domestic or gross national)).**ti,ab.**
Social reconnaissance.mp	‘Displacement (Psychology)’/	
Community-Based Participatory Research/	(displace$ adj2 (force$ or population or human or internal$)).mp	(lmic or lmics or third world or lami countr*).**ti,ab.**
**IKT**	Medical Missions, Official/	(low adj3 middle adj3 countr*).**ti,ab.**
Knowledge/	((relief or aid) adj2 work$).mp	transitional countr*.**ti,ab.**
(knowledge adj2 synthes*).mp.	exp war/	**
(knowledge adj2 translat*).mp.	War$1.mp	
(integrat* adj2 knowledg* adj2 translat*).mp.	(conflict affected adj3 (population$ or person$ or communit$ or state$)).mp	
(knowledge adj2 disseminat*).mp.	Avalanches/	
(knowledge adj2 exchang*).mp.	Earthquakes/	
Information Dissemination/	Floods/	
(information adj2 disseminat*).mp.	(avalanche$ or earthquake$ or flood or floods or flooding or flooded or landslide$ or tsunami$).mp	
engaged scholarship.mp.	Tidal Waves/	
stakeholder	Tsumanis/	
Translational Medical Research/	Cyclonic storms/	
Comparative Effectiveness Research/	(typhoon$ or hurricane$ or cyclone$).mp	
	Landslides/	
	Droughts/	
	drought$.tw.	
	Starvation/	
	(starvation or famine$).mp	
	(armed or zone) adj2 conflict$)).mp	
	Emergencies/	
	Emergency shelter/	
	Rescue work/	

**(Afghanistan or Albania or Algeria or Angola or Armenia or Armenian or Azerbaijan or Bangladesh or Benin or Byelarus or Byelorussian or Belarus or Belorussian or Belorussia or Belize or Bhutan or Bolivia or Bosnia or Herzegovina or Hercegovina or Botswana or Brasil or Brazil or Bulgaria or Burkina Faso or Burkina Fasso or Burundi or Urundi or Cambodia or Khmer Republic or Cameroon or Cameroons or Cameron or Camerons or Cape Verde or Central African Republic or Chad or China or Colombia or Comoros or Comoro Islands or Comores or Congo or Zaire or Costa Rica or Cote d’Ivoire or Ivory Coast or Cuba or Djibouti or Dominica or Dominican Republic or East Timor or East Timur or Timor Leste or Ecuador or Egypt or United Arab Republic or El Salvador or Eritrea or Ethiopia or Fiji or Gabon or Gabonese Republic or Gambia or Gaza or Georgia Republic or Georgian Republic or Ghana or Grenada or Guatemala or Guinea or Guiana or Guyana or Haiti or Honduras or India or Maldives or Indonesia or Iran or Iraq or Jamaica or Jordan or Kazakhstan or Kazakh or Kenya or Kiribati or Korea or Kosovo or Kyrgyzstan or Kirghizia or Kyrgyz Republic or Kirghiz or Kirgizstan or Lao PDR or Laos or Lebanon or Lesotho or Liberia or Libya or Macedonia or Madagascar or Malagasy Republic or Malaysia or Malaya Sabah or Sarawak or Malawi or Nyasaland or Mali or Marshall Islands or Mauritania or Mauritius or Agalega Islands or Mexico or Micronesia or Moldova or Moldovia or Moldovian or Mongolia or Montenegro or Morocco or Mozambique or Myanmar or Myanma or Burma or Namibia or Nepal or Nicaragua or Niger or Nigeria or Pakistan or Paraguay or Peru or Philippines or Philipines or Phillipines or Phillippines or Romania or Rumania or Roumania or Russia or Russian or Rwanda or Ruanda or Saint Lucia or St Lucia or Saint Vincent or St Vincent or Grenadines or Samoa or Samoan Islands or Sao Tome or Senegal or Serbia or Montenegro or Sierra Leone or Sri Lanka or Ceylon or Solomon Islands or Somalia or South Africa or Sudan or Suriname or Surinam or Swaziland or Syria or Tajikistan or Tadzhikistan or Tadjikistan or Tadzhik or Tanzania or Thailand or Togo or Togolese Republic or Tonga or Tunisia or Turkey or Turkmenistan or Turkmen or Uganda or Ukraine or USSR or Soviet Union or Union of Soviet Socialist Republics or Uzbekistan or Uzbek or Vanuatu or New Hebrides or Venezuela or Vietnam or Viet Nam or West Bank or Yemen or Zambia or Zimbabwe or Rhodesia).**hw,kf,ti,ab,cp.**

## Appendix B. Inclusion and exclusion criteria

**Table:**

Selection criteria	Yes/No
1. Does the full-text paper indicate health related research?	
2. Does the full-text paper indicate that participation occurred in one or more of the following three areas:	
a. partners were involved in identifying or setting the research questions?	
b. partners were involved in setting the methodology or collecting data or analysing the data?	
c. partners were involved in uptake or dissemination of the research findings?	
3. Does the full-text paper describe the research setting? (indicate community-based, organizational, or other (describe))	
a. humanitarian crises/humanitarian context/post-disaster context	
4. Does the full-text paper indicate empirical research (i.e. that there is some description of methods, data collection and analysis)? (Specify the methodology)	
5. Does the full-text paper describe PR-related outcomes?	
6. Does the full-text paper describe PR processes or contexts (or is there a reference to the process/context in a cited companion paper)?	

## Appendix C. Detailed description of scoping review steps

**Data sources**: The final search strategy included the following sources with publications since 2009: (1) electronic peer-reviewed health science databases (Medline and Embase), (2) an online database of published grey and peer-reviewed papers hosted by the humanitarian aid agency Médecins Sans Frontières (MSF), 3) databases from two humanitarian umbrella organisations ALNAP) and CHS, and 4) website resources or databases from seven of the bigger humanitarian organizations who had some kind of database with resources or academic publications (Action Contre la Faim (ACF), Care, International Rescue Committee (IRC), International Committee of the Red Cross (ICRC), Oxfam, Save the Children and World Vision).

**Inclusion and exclusion critera**: Exclusion criteria included: non-health-related humanitarian assistance, disaster preparedness, and studies that described a participative evaluation of humanitarian assistance. At the same time, we applied broad inclusion criteria for health-related assistance, meaning that all empirical studies related to the determinants of health (nutrition, poverty, etc.) or, for example, general needs assessments and water and sanitation studies were included. Nonetheless, studies on education and agriculture were excluded.

**Scanning process**: IO and SL met regularly throughout this phase to discuss the findings, adjust the selection criteria, and made decisions regarding the eligible studies for full review. We followed an adapted approach to the scoping review methodology as only one reviewer (IO) screened titles and abstracts, while all uncertainties were discussed with another team member (SL) (1). IO and SL independently assessed the full text of all (2) selected articles and together refined and agreed on the final inclusion and exclusion criteria. In this phase it was, for example, decided to include studies conducted in upper-middle-income countries, and to include studies conducted in humanitarian crisis settings (e.g. country in war) that were not directly related to humanitarian assistance. See Appendix C for the PRISMA flow-chart.

1. Pal NE, Eckenwiler L, Hyppolite S-R Ethical considerations for closing humanitarian projects: a scoping review. Journal of International Humanitarian Action. 2019;4(1):17.
2. Moher D, Liberati A, Tetzlaff J Preferred reporting items for systematic reviews and meta-analyses: the PRISMA statement. PLoS medicine. 2009;6(7):e1000097.
